# Development and validation of an equation to predict the incidence of coronary heart disease in patients with type 2 diabetes in Japan

**DOI:** 10.1186/s13104-021-05844-w

**Published:** 2021-11-25

**Authors:** Yasunari Yamashita, Gaku Inoue, Yoichi Nozaki, Rina Kitajima, Kiyoshi Matsubara, Takeshi Horii, Junichi Mohri, Koichiro Atsuda, Hajime Matsubara

**Affiliations:** 1 Division of Clinical Pharmacy (Laboratory of Pharmacy Practice and Science III), and Research and Education Center for Clinical Pharmacy, School of Pharmacy, Kitasato University, 9-1, Shirokane 5-Chome, Minato-ku, Tokyo, 108-8641 Japan; 2 AdvanceSoft Corporation, 4-3, Kandasurugadai, Chiyoda-ku, Tokyo, 101-0062 Japan; 3 Department of Pharmacy, Kitasato University Kitasato Institute Hospital, 9-1, Shirokane 5-Chome, Minato-ku, Tokyo, 108-8641 Japan; 4 Division of Clinical Pharmacy (Laboratory of Pharmacy Practice and Science I), and Research and Education Center for Clinical Pharmacy, School of Pharmacy, Kitasato University, 1-15-1 Kitasato, Minami Ward, Sagamihara, Kanagawa 252-0375 Japan; 5 Department of Pharmacy, Kitasato University Hospital, 1-15-1 Kitasato, Minami Ward, Sagamihara, Kanagawa 252-0375 Japan

**Keywords:** JJ risk engine, Type 2 diabetes, Coronary heart disease, Discrimination, Calibration, Hypoglycemia, C-statistic, Box plot, Outlier

## Abstract

**Objective:**

In the diabetes treatment policy after the Kumamoto Declaration 2013, it is difficult to accurately predict the incidence of complications in patients using the JJ risk engine. This study was conducted to develop a prediction equation suitable for the current diabetes treatment policy using patient data from Kitasato University Kitasato Institute Hospital (Hospital A) and to externally validate the developed equation using patient data from Kitasato University Hospital (Hospital B). Outlier tests were performed on the patient data from Hospital A to exclude the outliers. Prediction equation was developed using the patient data excluding the outliers and was subjected to external validation.

**Results:**

By excluding outlier data, we could develop a new prediction equation for the incidence of coronary heart disease (CHD) as a complication of type 2 diabetes, incorporating the use of antidiabetic drugs with a high risk of hypoglycemia. This is the first prediction equation in Japan that incorporates the use of antidiabetic drugs. We believe that it will be useful in preventive medicine for treatment for people at high risk of CHD as a complication of diabetes or other diseases. In the future, we would like to confirm the accuracy of this equation at other facilities.

**Supplementary Information:**

The online version contains supplementary material available at 10.1186/s13104-021-05844-w.

## Introduction

It is important to prevent the development of diabetic complications during diabetes treatment [1]. One of the methods to predict the incidence of diabetic complications is through the use of risk engine, which is used to personalize medicine for patients. Currently, several risk engines have been developed to predict the incidence of diabetic complications [1–5]. In 2012, the Japan Diabetes Complications Study (JDCS)/The Japanese Elderly Diabetes Intervention Trial (J-EDIT) risk engine (JJ risk engine) was developed to accurately predict macro- and microvascular complications in Japanese patients with type 2 diabetes [1]. However, after the Kumamoto Declaration 2013, this risk engine was evaluated only through internal validation, without considering hypoglycemia prevention as the priority, and the implementation of external validation has been a challenge [1, 6]. In our previous study, we externally validated the prediction accuracy of the JJ risk engine using data from patients with type 2 diabetes at Hospital A. The results showed that the prediction of the JJ risk engine and the actual frequency of diabetic complications in Hospital A diverged [7]. Although the cause of this discrepancy is unknown, one reason may be the change in diabetes treatment to one that emphasizes hypoglycemia prevention [6, 8–10]. Therefore, we conclude that it is difficult to accurately predict the complication rates in patients using the JJ risk engine based on the diabetes treatment policies after the Kumamoto Declaration of 2013 [7].

Besides hypoglycemia, other risk factors for coronary heart disease (CHD) include aging, hypertension, hyperlipidemia, obesity, and chronic kidney disease (CKD) [11, 12]. Outlier tests were conducted for each risk factor, because prediction without outliers is more accurate than prediction with outliers [13, 14]. Therefore, in this study, we developed a new prediction equation that is more accurate and suitable for the patient population of Hospital A and externally validated the new prediction equation using patient data from Hospital B.

## Main text

### Methods

#### Hospital (target facility)

Kitasato University Kitasato Institute Hospital (Hospital A).

Kitasato University Hospital (Hospital B).

### Patients

#### Selection criteria

The subjects were patients with type 2 diabetes who visited Hospital A or Hospital B from January 2013 to December 2013 and continued treatment for the following 5 years until 2018.

### Exclusion criteria

Patients who refused to participate in the study or had a history of any of the following diseases were excluded: angina, myocardial infarction, stroke, peripheral arterial disease, familial hypercholesterolemia, familial type III hyperlipidemia, nephrotic syndrome, renal diseases other than diabetic nephropathy, microhematuria, pre-proliferative and proliferative retinopathy, or major ocular diseases (e.g., glaucoma, dense cataract, or a history of cataract surgery).

This study was conducted in accordance with the Ethical Guidelines for Medical and Health Research Involving Human Subjects. The Kitasato University Kitasato Institute Hospital, Research Ethics Committee, approved the study (Control Number: 20051 and 20051-2) and provided permission to review patient records and use the corresponding data. The option to opt-out of the study was provided to the patients at the start of the study (2021).

### Statistical analysis

We developed a prediction equation based on the Cox proportional hazard model using patient data from Hospital A [15]. The backward stepwise method was used for the selection of variables [16].

### Discrimination

It is an index that evaluates how accurately the presence or absence of an event can be predicted by a prediction model. The C-statistic, which is calculated based on the receiver operating characteristic (ROC) curve, is used as a criterion for measuring the predictive accuracy [17, 18].

### Calibration

It is an index to measure the degree of agreement between the prediction by the model and the actual outcome. The significance probability calculated using the Hosmer–Lemeshow test is used as the criterion for predictability. The significance level was set at 0.05 (p < 0.05) [18, 19].

### External validation

We developed prediction equations using Hospital A data and then performed external validation using Hospital B data.

### Outlier testing using box plots

Outlier tests with box plots were performed to reduce the impact of outliers of each risk factor on the prediction accuracy.

A box-and-whisker diagram of data for age, total cholesterol, HDL cholesterol, body mass index (BMI), urinary albumin, upper quartile (Q3), and lower quartile (Q1) was generated, and values that deviated from the range of Q1–(Q3–Q1) to Q3 + (Q3–Q1) were considered outliers [20].

Although blood pressure was measured at the time of medical examination, it was excluded from the risk factors in this study because the time of measurement varied among subjects [21].

R version 2.5.1 (http://www.r-project.org, library Design, Hmisc, ROCR) was used to determine discrimination and calibration, whereas the ROC curve, Hosmer–Lemeshow test, and box plot were used for calculation [18–20].

## Results

There were 572 and 285 patients in Hospitals A and B, respectively. The baseline characteristics of the patients are presented in Table 1.

**Table 1:** Baseline characteristics

	Kitasato University Kitasato Institute Hospital			Kitasato University Hospital		
	Hospital A			Hospital B		
Characteristics	All patients (n = 572)	Male (n = 384)	Female (n = 188)	All patients (n = 285)	Male (n = 169)	Female (n = 116)
Age (years)	64.5 ± 10.5	63.9 ± 10.5	65.6 ± 10.5	62.3 ± 12.4	61.0 ± 12.5	64.3 ± 12.0
BMI (kg/m^2^)	25.0 ± 4.3	25.0 ± 4.0	24.1 ± 4.2	25.9 ± 4.8	26.1 ± 4.4	26.4 ± 5.5
Simple retinopathy, n (confirmed/total)	46/572	35/384	11/188	81/285	41/169	40/116
Atrial fibrillation, n (confirmed/total)	10/572	8/384	2/188	9/285	4/169	5/116
Presence of exercise habits, n (confirmed/total)	91/572	63/384	28/188	17/285	14/169	3/116
Current smoker, n (confirmed/total)	84/572	66/384	18/188	41/285	31/169	10/116
Disease duration (years)	12.0 ± 8.3	12.7 ± 8.6	10.6 ± 7.6	13.1 ± 10.9	13.1 ± 10.8	13.0 ± 11.0
HbA1c (NGSP%)	7.3 ± 2.8	7.3 ± 3.3	7.1 ± 0.8	8.1 ± 1.8	8.0 ± 1.8	8.2 ± 1.8
Systolic blood pressure (mmHg)	126 ± 14	125 ± 14	126 ± 13	132 ± 15	130 ± 14	134 ± 15
Total cholesterol (mg/dL)	189 ± 31	184 ± 30	198 ± 30	198 ± 37	203 ± 39	192 ± 34
HDL cholesterol (mg/dL)	63 ± 17	60 ± 17	69 ± 17	58 ± 16	56 ± 17	60 ± 15
Urinary albumin (mg/gCr)	66 ± 245	65 ± 239	69 ± 257	186 ± 593	215 ± 684	143 ± 423
LDL cholesterol (mg/dL)	107.4 ± 26	105.4 ± 26	112.1 ± 25	–	–	–
Medicine [sulfonylurea (SU) and/or insulin], n (confirmed/total)	338/572	239/384	99/188	195/285	115/169	80/116
SU, n (confirmed/total)	252/572	276/384	76/188	67/285	40/169	27/116
Insulin, n (confirmed/total)	104/572	79/384	25/188	131/285	76/169	55/116

Data are expressed as means ± standard deviation

Patients who used either sulfonylurea (SU) drugs or insulin were considered medicine users. Among the variables, only medicine was found to have a value of p < 0.05 (p = 0.03) (Additional file 1: Table S1). Therefore, only medicine was included as a variable in the prediction equation, and the prediction equation developed is as follows:

$$ \left[ {{\text{K}}{-}{\text{medicine equation}}} \right] $$

$$ \lambda_{{\text{t}}} \, = \,\lambda_{{0{\text{t}}}} \, \times \,{\text{exp }}\left\{ {\beta \, \times \,{\text{medicine }}\left( {0,{ 1}} \right)} \right\} $$

λ_t_: Incidence rate by time t; λ_0t_: Baseline hazard for time t; β: partial regression coefficient; medicine (0, 1): 1 for patients who used either SU or insulin, 0 for patients who used neither.

The prediction equation using data from Hospital A resulted in a C-statistic of 0.734 and a calibration of p > 0.05, indicating no significant difference between the measured and predicted values. In contrast, external validation using data from Hospital B resulted in a C-statistic of 0.809 and a calibration of p < 0.05, indicating a significant difference between the measured and predicted values (Table 2).

**Table 2:** Development and external validation of prediction equations

	n	β	HR	Discrimination; C-statistic (95% CI)	Calibration
Hospital A	572	1.39	4.0 (1.2–13.7)	0.734 (0.630–0.839)	0.292
Hospital B	285	–	–	0.809 (0.721–0.897)	0.006

Therefore, an outlier test using a box-and-whisker diagram was performed to improve the prediction accuracy. The outliers were age: 39 years or less; total cholesterol: ≥ 266 and ≤ 96; HDL cholesterol: ≥ 105.5; BMI: ≥ 35; and urine albumin: ≥ 78.7. A total of 120 patients from Hospital A and 84 patients from Hospital B were excluded.

In the analysis of the variables after exclusion, the p-value for medicine was < 0.05, indicating a significant difference.

The developed prediction equation is as follows:

$$ \lambda_{{{1825}}} \, = \,0.0{1}\, \times \,{\text{exp }}\left\{ {{1}.{73}\, \times \,{\text{medicine }}\left( {0,{ 1}} \right)} \right\} $$

λ_1825_: Incidence rate within 5 years; medicine (0, 1): 1 for patients who used either SU or insulin, 0 for patients who used neither.

The C statistic was 0.644 and the calibration was p > 0.05. There were no significant differences between the measured and predicted values. A total of 201 excluded patients from Hospital B were used for external validation; the C statistic was 0.750, and the calibration was p > 0.05. There were no significant differences between the measured and predicted values (Table 3).

**Table 3:** Development and external validation of prediction equation after outlier testing

	n	β	HR	Discrimination; C-statistic (95% CI)	Calibration
Hospital A	452	1.73	5.6 (1.3–24.8)	0.644 (0.523–0.758)	0.974
Hospital B	201	–	–	0.750 (0.651–0.849)	0.322

## Discussion

After the outliers were excluded, a prediction equation (K-medicine equation) was developed for the incidence of CHD in patients with type 2 diabetes using patient data from Hospital A. Furthermore, after excluding outliers, the external validation using data from Hospital B showed that the C-statistic was moderate and the calibration was not significantly different, indicating a correct prediction. Exclusion of outlier data for age, total cholesterol, HDL cholesterol, BMI, and urinary albumin levels was a condition used in this prediction equation.

Compared to the JJ risk engine, we incorporated the use of antidiabetic drugs into the prediction equation. In addition, while risk engines developed in other countries have incorporated therapeutic drugs (dyslipidemia drugs) into the prediction equation [22], our study is the first to incorporate therapeutic drugs (diabetes drugs) into the prediction equation in Japan. This prediction equation is intended for the primary prevention of CHD in Japanese patients with type 2 diabetes. Compared with other prediction formulae, this equation has the advantage that the variables can be selected according to the characteristics of each institution; the disadvantage is that the risk of developing CHD is calculated simultaneously prior to the administration of SU drugs and insulin, because SU drugs and insulin are introduced much later in diabetes treatment.

In contrast, validation using Hospital B patient data showed significant differences in the calibration, indicating an incorrect prediction. As per previous studies [11–14], the incorrect prediction might be owing to outliers of risk factors that influence the development of CHD, which affect the prediction accuracy.

In a previous study, the risk of developing CHD was higher in patients who used SU drugs and insulin than in those who received dipeptidyl peptidase-4 inhibitors [23], indicating that SU drugs and insulin are risk factors for CHD.

In the selection of variables, there was a significant difference in the presence or absence of SU drugs or insulin use, and it was reasonable to include it in the variables of the prediction equation. The risk of CHD associated with the use of SU drugs and insulin is consistent with the results of previous studies [23]. This prediction equation indicates that the use of diabetes medications with a high risk of hypoglycemia influences the development of CHD as a complication of type 2 diabetes.

Because of outliers, about 21% and 30% of patients were excluded in Hospital A and Hospital B, respectively. As more than 70% of the patients in each institution remained after exclusion, we believe that our prediction formula is applicable to many patients with type 2 diabetes.

In the variable analysis, there was no significant difference in the risk factors for CHD. Therefore, in this study, the risk factors for CHD were not included as a variable in the prediction equation; however, for patients with elevated laboratory values for risk factors for CHD, it will need to be considered as risk in the future. The column for LDL cholesterol under Hospital B (Table 1) is blank, because the LDL cholesterol values could not be obtained at this hospital. Since the blood pressure data presented in this study is only what was collected at the clinic, it is necessary to collect blood pressure data multiple times at the time of consultation and conduct additional analysis using more reliable blood pressure data [21].

The unique feature of this study is that the prediction equation was developed using patient data from a medium-sized hospital in Japan, rather than large-scale clinical data. Furthermore, we were able to create a prediction equation and validate externally. Although this prediction equation focuses on drugs that tend to cause hypoglycemia, which is a risk factor for the development of CHD, some hypoglycemic drugs, such as SGLT2 inhibitors and GLP-1 receptor agonists, reduce the risk of CHD [24]. In Hospital A, we have been using these drugs at full scale since 2016. Less than 9% of patients use these drugs; and therefore, it is difficult to consider the impact of these drugs in this study. The frequency of use of these drugs is expected to increase in the future; and it is necessary to consider the development of a prediction formula that includes them.

## Conclusion

We developed an equation to predict the incidence of CHD in patients with type 2 diabetes. Based on the prediction equation developed in this study, we believe that the use of diabetic drugs with a high risk of hypoglycemia influences the incidence of CHD as a complication of type 2 diabetes. Although this prediction equation is based on the patient population of Hospital A, we would like to confirm the accuracy of our prediction theory in other institutions in the future.

## Limitations

The analysis of this study included patients who were using medications to prevent cardiovascular disease, but the effects of these medications were not considered in the analysis. Consequently, the possibility that they may affect the outcome cannot be excluded. A history of CHD in the family could influence a patient's risk of developing CHD. However, in this study, we were unable to investigate family history, which could affect the results of the prediction equation. Therefore, family history should be considered in future studies.

The relatively small number of people who developed CHD may have affected the reliability of the analysis [25].

## Supplementary Information

**Additional file 1: Table S1.** Analysis results for variables.

## Data Availability

The datasets generated and analyzed during the current study are not publicly available owing to the privacy of the research participants. Data are available from the corresponding author upon reasonable request; however, permission from the Kitasato University Kitasato Institute Hospital Research Ethics Committee is required.

## Abbreviations

CHD: Coronary heart disease
ROC: Receiver operating characteristic
HbA1c: Glycated hemoglobin
NGSP: National Glycohemoglobin Standardization Program
HR: Hazard ratio
SU: Sulfonylurea
LDL: Low-density lipoprotein
