# Supplementary material for: Development and validation of an equation to predict the incidence of coronary heart disease in patients with type 2 diabetes in Japan
Source: BMC Res Notes. 2021 Nov 25;14:426. doi: 10.1186/s13104-021-05844-w (PMC8613942; doi:10.1186/s13104-021-05844-w)
Supplement: Supplementary file 1 — Additional file 1: Table S1. Analysis results for variables. [file 13104_2021_5844_MOESM1_ESM.docx]

Additional file: Table S1 Analysis results for variables

| Variable | p-value |
| --- | --- |
| Sex | 0.23 |
| Age (years) | 0.16 |
| BMI | 0.89 |
| Simple retinopathy | 0.62 |
| Atrial fibrillation | 0.27 |
| Presence of exercise habits | 0.62 |
| Current smoker | 0.51 |
| Disease duration | 0.28 |
| HbA1c (NGSP,%) | 0.52 |
| Systolic blood pressure (mmHg) | 0.49 |
| Total cholesterol (mg/dL) | 0.84 |
| HDL cholesterol (mg/dL) | 0.32 |
| LDL cholesterol (mg/dL) | 0.86 |
| Urinary albumin (mg/gCr) | 0.63 |
| Medicine | 0.03* |
| Glinide | 1.00 |
| Dipeptidyl-peptidase IV inhibitor | 0.86 |
| GLP-1 receptor agonist | 1.00 |
| Biguanide | 0.73 |
| Thiazolidinediones | 0.81 |
| Alpha-glucosidase inhibitor | 0.36 |

*: p < 0.05
